# Comparison of the CBA-H and SF-36 for the screening of the psychological and behavioural variables in chronic dialysis patients

**DOI:** 10.1371/journal.pone.0180077

**Published:** 2017-06-30

**Authors:** Concetta De Pasquale, Daniela Conti, Maria Luisa Pistorio, Pasquale Fatuzzo, Massimiliano Veroux, Santo Di Nuovo

**Affiliations:** 1 Vascular Surgery and Organ Transplant Unit, Department of Medical, Surgery Sciences and Advanced Technologies “G.F. Ingrassia”, University of Catania, Catania, Italy; 2 Department of Educational Sciences, University of Catania, Catania, Italy; 3 Faculty of Arts, Computing, Engineering and Science, Sheffield Hallam University, Sheffield, United Kingdom; 4 Department of Medical and Paediatric Sciences, University of Catania, Catania, Italy; Universidade Estadual Paulista Julio de Mesquita Filho, BRAZIL

## Abstract

The aim of the study was to perform an analysis of the emotional reactions, perception of stressful life and behavioural changes related to Haemodialysis (HD) in order to identify those variables that can improve lifestyle and the adherence to treatment. Some psychometric assessment, such as the Cognitive Behavioural Assessment, Hospital Form, (CBA-H) and the Health Survey (SF-36), which provides two indexes: the Physical Component Score (PCS) and the Mental Component Score (MCS), are suitable to assess a patient’s psychological and behavioural style and their health-related quality of life. The study involved 37 Italian out-patients with end-stage renal disease under HD therapy. We calculated the Spearman correlation between variables of CBA-H, SF-36, age and time on HD. We also performed a multivariate linear regression using the CBA-H variables as predictors and PCS and MCS as dependent variables. From the CBA-H, 95% of participants self-reported psychological characteristics comparable to Type A personality, which identifies an anxious, hyperactive and hostile subject. Physical limitations were found to be directly proportional to the time on dialysis (**r**_**s**_ = -0.42). The condition of perceived stress worsens the state of mental health (**r**_**s**_ = -0.68) and general health perception (**r**_**s**_ = -0.44). The condition of vital exhaustion correlates both the PCS and the MCS (p<0.01) with possible outcomes of physical and mental illness. The psychological wellbeing of a dialyzed patient could be due to the combination of several factors, including life parameters, the positive perception of psychosocial outcomes, and the perceived quality of life. A multidisciplinary team (neurologists, psychiatrists, psychologists, and nurses) is essential to plan effective psychological and psychotherapeutic interventions to improve a mind-body integration.

## Introduction

Any pathological event that affects a person produces important emotional, psychological and social reactions. Differently from other chronic illnesses, the Haemodialysis (HD) patients are in an unusual existential condition. The condition of dependency in daily living activities is coercive and becomes a source of serious frustrations. If, on one hand, the dialysis gives the patients a strong reassurance of survival, on the other hand it may also suggest the certainty that the state of the distress will last forever [1].

These conditions can cause severe intrapsychic problems, unpredictable aggressive reactions and rage but even more with self-directed, self-harming behaviour. In literature, many studies are concerned with the comorbid psychopathology in dialysis patients, mostly regarding depressed mood, anxious and/or psychotic demonstrations and the perception of stress [2–6]. Moreover, despite Chronic Kidney Disease (CKD) being associated with a low perception of health status and an alteration of Quality of Life (QL), studies confirm that acceptance of the disease contributes to the improvement of physical and mental components of self-reported health [7,8].

Furthermore, the different types and styles of behaviour are related to the pathological severity of the event and also to the related stress and frustration tolerance. For this reason, Friedman and Rosenman [9] defined Type A and Type B personalities on the basis of the different responses to stressful situations. The “Type A Personality” (TAP) predisposes to cardiovascular disorders, and characterizes individuals who are in a constant state of tension, hyperactive, and with a tendency to be hostile. The latter condition occurs when the subject has the feeling of not being able to have control, either of their own actions or on their social environment [10]. By contrast, “Type B” people are more relaxed and they adapt to their own experiences of stressful life with less tension and more acceptance, experiencing positive emotions. This personality construct was studied in relation to Ischemic Heart Disease [11–14]; but some studies (e.g. [15]) questioned whether it was predictive of specific pathologies. However, it is widely accepted as a reliable personality construct, and has been integrated in several methods of assessment, including the Minnesota Multiphasic Personality Inventory (MMPI-2).

This construct is relevant to HD, which is a condition of stress involving both a care condition and a threat to the psychological wellbeing of person [16,17], inducing tension and latent hostility. The thinking of nephropathic and haemodialysis patients is mainly centred on the disease, causing tension and latent hostility towards themselves, their family members, and healthcare workers. The future appears uncertain to them and this causes them to be poorly cooperative, with alternating periods of closing and loss of control.

In light of the above, the aim of this study was to analyse the patterns of personality that, even though they are durable and stable, in conditions of subjective distress or organic disease, can become maladaptive and adversely affect the ability of a subject to regulate their emotions and state of physical and mental wellbeing. These variables can change the style of life and adherence to treatment.

In the present study, we hypothesized that the general quality of life in HD subjects correlates with some personality characteristics typical of the Type A cluster, and with some demographic characteristics of the sample: Age (A), Education (E), Marital Status (MaS), Male Gender (MG) and Time on Haemodialysis, expressed in months (TH).

## Materials and methods

### Study sample

The study involved Italian patients with end-stage renal disease (ESRD), who were receiving ambulatory HD therapy in private centres for dialysis. Participants were recruited by the Organ Transplant Unit and the selection was based on a number of criteria: that they had started the haemodialytic treatment of renal function replacement at least 3 months before; were at least 20 years old; had no previous transplant experience, and were without psychiatric disorders. The basic psychiatric evaluation was performed using two assessment scales of transverse symptoms, level I and II of DSM 5, to examine any critical psychopathological domains. This made it possible to exclude the presence of psychiatric disorders according to the Diagnostic and Statistical Manual for Mental Disorders, 5th Edition (DSM 5) or concomitant use of psychiatric drugs that could influence cognitive and emotional aspects [18]. The response rate was 100%.

Before the study began, it was reviewed and approved by the ethical committee of the School of Medicine of the University of Catania. All patients involved gave their written, informed consent before being included in the study.

The research involved the evaluation of 37 patients with ages between 20 to 75 years old (M = 52.16 ±13.40).

### Measures

Personality and behavioural style were assessed using the Cognitive Behavioural Assessment, Hospital Form (CBA-H).

The questionnaire CBA-H is composed of 152 dichotomous items which are easy to read and understand, and which are organized into four cards (A, B, C and D).

Card A (21 items) investigates Anxiety (A) Health-care related fears (HF) and Depression (D).

Card B (23 items) examines Emotional instability-Depressive mood (ED), Psychophysical Wellbeing (PW), and Perceived psychophysical Stress (PS) concerns in the previous three months.

Card C (61 items) allows the patient to describe his or her general character and behaviour. The items analyse stable traits and characteristics, e.g. Neuroticism (N), Social Anxiety (SA), Haste and Impatience (HI), Excessive Involvement (EI), Hostility (H), Inability to Relax (IR), Interpersonal Difficulties (ID), Leadership/Competitiveness (LC) and Irritability (I). The combination of Haste and Impatience, Excessive Involvement, Hostility, Inability to Relax, Leadership/Competitiveness and irritability quantifies the presence of a Type A personality.

Card D (47 items) explores the lifestyle and possible health risk factors (stressful events, smoking, eating and drinking, quality of sleep etc.) with changes in behaviour related to the evolution of the disease [19].

The CBA-H questionnaire also gives a score of Vital Exhaustion index (VE), composed of Interpersonal Difficulties (ID), and Perceived psychophysical Stress (PS) [10,20]. All CBA-H scores are in a negative direction (i.e. the higher the score, the higher the reported pathology).

The concordance between the CBA-H scores and clinical judgment of the psychologist was tested in 4888 patients with chronic diseases (heart disease, lung disease, neoplastic diseases and degenerative diseases of the central nervous system).

The CBA-H manual [20] shows the descriptive statistics (Cronbach's alpha value, and cut-off value of the individual scales). The CBA-H hasn’t been validated on haemodialysis patients.

The quality of life was studied through the Complete Form Health Survey (SF-36), a patient-reported survey of patient health. It consists of eight subscales reporting scores between 0 and 100: lower scores indicate greater disability. The validity and reliability of SF-36 has been confirmed in patients with end-stage renal disease [21] and in kidney transplant recipients [22]. The considered variables in this study were: Physical Functioning (PF), Bodily Pain (BP), General Health Perception (GHP), Physical Role Functioning (PrF-physical problems), Emotional role Functioning (EF-emotional problems), Vitality (V), Social role Functioning (SF), and Mental Health (MH). It then produces summary measures, i.e. a Physical Component Score (PCS) and a Mental Component Score (MCS) [23].

Assessments were administered as part of the psychological and psychiatric assessment required for inclusion into the waiting list for kidney transplant.

### Statistical analyses

Each variable of CBA-H was compared with the standardization sample of the Italian population [20] and the values over the cut-off of 75° percentile were computed, with a preliminary aim of indicating the relevance of the different psychological problems in our dialysis sample. All the participants were ranked in order, and the percentages of subjects over the cut-off for each variable were reported.

For the variables of the SF-36 questionnaire—according to the different criteria of standardization for the test [24]—the cut-off was set at two standard deviations under the mean (corresponding to a score of 30 out of 100). Also in this case, the percentages of subjects under the cut-off were listed in order according to the relevance of the problem in the sample.

This descriptive analysis of the general relevance of the problematic areas in the dialysed sample did not influence the subsequent analyses, which were made on the whole sample.

We conducted a nonparametric statistical test on PCS, MCS and TAP variables to investigate whether gender, education, time on dialysis and marital status influenced these constructs. We performed a three-way ANOVA on ranks (Type III Sum of Squares).

Furthermore, correlation analyses were performed using the Spearman technique to determine how the variables of CBA-H and SF-36 would relate to age and time on haemodialysis. Then, we calculated the Spearman’s *r*_*s*_ correlation between the CBA-H and the SF-36 score. For these analyses, the CBA-H variables were all rotated at the negative pole, while the SF-36 all to the positive pole.

We also performed multivariate linear regressions using the CBA-H variables as predictors and PCS and MCS as dependent variables. The Stepwise method, backward (to-remove p = .15) was used.

The Statistical Package for Social Sciences (SPSS) version 24 was used for statistical analyses.

## Results

### Details of participants

Of the 37 patients diagnosed with terminal kidney disease, 70.3% were male and 29.7% were female; 40% lived in urban environments (≥100.000), while 60% lived in rural environments (<100.000). Regarding their level of education, 76% had no more than eight years of schooling, whereas 24% had been educated for between nine and twelve years. Concerning their profession, 65% were housewives, unemployed or retired and 35% were employed.

Of the surveyed subjects, 35% were single (24% unmarried and 11% divorced) and 65% were part of a couple.

Regarding the original disease, hypertensive nephrosclerosis (no biop.) (49%) was the most common cause of chronic renal failure, followed by diabetic nephropathy (19%), chronic glomerulonephritis (12%) and polycystic kidney disease (8%). All patients were dialyzed 3 times a week for 3 or more hours each time. The quality of dialysis was good, showing 86.5% of patients with a Kt/V ≥ 1.2. In 41% of the cases phosphatemia levels were under 5.5 mg/dL; 54% had albumin levels over 4 g/dL and 51% had haemoglobin higher than 10 g/dL.

The socio-demographic and clinical data of these patients is summarized in Table 1.

**Table 1:** Descriptive data for patients on haemodialysis.

Characteristics:	Participants (n = 37)	Frequencies
Age, mean ± SD (range)	52.16 ± 13.40	
20–40	14%	5
41–50	24%	9
51–59	38%	14
60–69	19%	7
70+	5%	2
Male gender	70.3%	26
Female gender	29.7%	11
Time on haemodialysis (months)	20.38 ± 22.45	
**Original disease**:		
Hypertensive nephrosclerosis	49%	18
Diabetic nephropathy	19%	7
Chronic glomerulonephritis	12%	4
Polycystic kidney disease	8%	3
Unknown	13%	5
Kt/V	≥ 1.2	

The study of psychological and behavioural variables through the CBA-H showed that 95% of the dialyzed sample had the psychological traits comparable to those of a Type A Personality, characterized by impatience (59%), hyperactivity (70%), hostility (24%) and a tendency towards competitiveness (8%). Other psychic symptoms which emerged were irritability (41%), changes in mood (32%) and anxiety (30%), all of which are expressions of a difficult psycho-organic condition.

The study of the Quality of Life through the SF-36 test showed that 49% of HD patients manifested limitations in their physical capability, and 27% experienced limitations in their emotional capability.

According to the ANalysis Of VAriance (ANOVA) education (E), time on haemodialysis (TH) and marital status (ST) do not influence the Physical Component Score (E = .76; TH = .49; MS = .72), the Mental Component Score (E = .43; TH = .17; MS = .71) and Type A Personality (E = .91; TH = .09; MS = .53) but the latter is significantly influenced by the male gender (p = .05).

The Spearman correlation between CBA-H and SF-36 variables and increasing haemodialysis time (shown in Table 2) shows the patients' tendency to get used to the treatment. Indeed, the ability to relax increases, while the physical pain and the perception of physical function decrease. As regards the correlation with age, there is evidence of increased Hostility and Excessive Involvement, while the psychological characteristics of a Type A Personality, especially the excessive involvement and impatience, are more evident as the age of the subjects on dialysis increases.

**Table 2:** Spearman correlation between age, time on haemodialysis and variables of CBA-H and SF-36.

	Age	Time on haemodialysis (months)
**Scale CBA-H (negative scores)**		
Anxiety	.00	-.02
Health-care related fears	-.03	.10
Depression	.08	.23
Emotional instability-Depressive mood	-.02	.22
Psychophysical Wellbeing	.00	-.22
Perceived psychophysical Stress	-.13	-.17
Neuroticism	-.07	-.16
Social Anxiety	.19	.20
Haste and Impatience	.28*	.09
Excessive Involvement	.35*	.26°
Hostility	.13	.31*
Inability to Relax	-.21	-.27°
Interpersonal Difficulties	.14	.17
Leadership/Competitiveness	.02	.05
Irritability	.05	-.23
Type A Personality	.50***	.23
Vital Exhaustion	-.15	-.12
**SF-36 (positive scores)**		
Physical Functioning	-.04	-.42**
Physical role Functioning	.17	-.14
Bodily Pain	.18	-.34*
General Health Perceptions	.10	.08
Vitality	.20	-.17
Social role Functioning	-.02	.09
Emotional role Functioning	.01	.07
Mental Health	.20	.08
Physical Component Score	.16	-.29°
Mental Component Score	.10	.16

Significance °p < .10 *p<0.05 **p<0.01 ***p<0.001

Significant correlations between the scales of the CBA-H and SF-36 are shown in Table 3.

**Table 3:** Correlations (*r*_*s*_ Spearman) between CBA-H scores (all the negative pole) and the quality of life scores (SF-36).

	PF	PrF	BP	GHP	V	SF	EF	MH
**Anxiety**	-.02	-.34*	-.12	-.29	-.31	-.21	-.60	-.58
**Health-care related Fears**	-.32*	-.23	-.16	-.31	-.29	-.24	-.23	-.46*
**Depression**	-.25	-.27	-.10	-.18	-.21	-.13	-.17	-.36*
**Psychophysical Wellbeing**	.19	.31	.24	.43	.38*	.13	.15	.40*
**Social Anxiety**	-.17	-.02	-.12	-.45	-.33*	-.21	-.39*	-.52
**Excessive Involvement**	-.09	-.32*	-.04	.04	.03	.03	-.08	.17
**Hostility**	-.13	-.39*	-.15	-.05	-.29	-.22	-.38*	-.30
**Interpersonal Difficulties**	-.22	-.15	-.23	-.11	-.25	-.21	-.42	-.40*

Significance r_s_ *p<0.05

Abbreviations: PF, Physical Functioning; PrF, Physical role Functioning; BP, Bodily Pain; GHP, General Health Perceptions; V, Vitality; SF, Social role Functioning; EF, Emotional role Functioning; MH, Mental Health.

Specifically, the variable Health-care related Fears (HF), which is the situation of distress experienced during medical visits and the frequent clinical examinations to which a haemodialysis patient is exposed, refers to a condition of dependence and uncertainty given by the disease condition and correlates negatively with Physical Functioning (PF) and Mental Health (MH). Interestingly, furthermore that psychological variables of Type A Personality (TAP), do not change with their own General Health Perceptions (GHP) to the exclusion of Hostility (H) which is most evident with increasing both the Physical role Functioning (PrF) and Emotional role Functioning (EF).

Conversely, Psychological Well-being (PW) directly correlates to the Mental Health (MH) and Vitality (V), while Mental Health (MH) correlates negatively to Depression (D) and Interpersonal Difficulty (ID). Furthermore, the condition of Social Anxiety (SA) and Hostility (H) worsen the Mental Health (MH) and Emotional Role Functioning (EF).

Finally, we considered it important to verify if the condition of Vital Exhaustion (VE), related to the mood (ED) and Perceived Stress (PS), situations of Interpersonal Difficulties (ID) and social disadvantages (SA) experienced by haemodialysis patients could influence their own physical and mental health with possible physical and psychological organic consequences.

In order to evaluate the degree of prediction of the variables in the Mental Component Score (MCS) and Mental Component Score (MCS) seventeen linear regressions were performed.

For Physical Component Score (PCS) we used the backward method (to-remove p = .15. Multiple R = 0.54; Squared Multiple R = 0.29) and significant predictors have been identified: Vital Exhaustion (*r*2 = -.710, *p* = .001), Neuroticism (*r*2 = .381, *p* = .042) and Social Anxiety (*r*2 = .256, *p* = .137).

For the significant predictors of the Mental Component Score (MCS) we also used the backward method (to-remove p = .15. Multiple R = 0.74; Squared Multiple R = 0.55) and identified: Vital Exhaustion (*r*2 = -.496, *p* = .004) and Anxiety (*r*2 = -.305, *p* = .063).

Specifically, the condition of Vital Exhaustion (VE), characterized by Depressive mood (ED) and Perceived psychophysical Stress (PS), had a negative influence on the condition of physical and mental wellbeing (PW), self-perceived by the haemodialysis patient. Furthermore, the Anxiety state (A) on the haemodialysis condition produces negative effects on their mental health perception (MCS).

Moreover, social hardship and the tendency to experience somatization are positive predictors of psychological wellbeing because they increase the activation of attitudes, cognitions and behaviours that can determine the evolution and the resilient adaptation to a “different health” condition.

## Discussion and conclusions

For Chronic Kidney Disease (CKD) patients the haemodialysis therapy is on one hand separate from the threat of danger to life and the anguish of death; on the other hand, it imposes a psychic suffering (distress) for continuous effort to adapt to unusual existential conditions with strong limitations and practical difficulties in everyday life [3–5]. The thoughts of the HD patient mainly concern, sometimes obsessively, the disease with somatization and a tendency toward hypochondria [2]. The patient tends to be self-centred, indifferent to the needs of others, basically anxious and emotionally unstable, all typical variables of Type A Personality [11].

The psychological wellbeing of HD patients depends not only on physical health, but also on the positive perception of psychosocial variables and especially the quality of life [7,25,26]. If the HD therapy on one hand reduces the threat of danger to life and consequently the fear of death, on the other hand it can impose a psychic distress due to the continuous effort to adapt to unusual existential conditions, with strong limitations and practical difficulties in everyday life [3–5].

In HD patients an enhanced vital exhaustion, resulting in a poor "life energy", exceeds the need to control every aspect of their disease in order to achieve the best possible life balance.

Moreover, with regard to the overall functioning of the HD patient, we can hypothesize that the predominant psychological symptoms could have consequences on the quality of their relationships. These conditions can hinder the development of friendships and also result in conflicts in family relationships.

In light of this, we considered it essential to underline the consciousness of the psychological and existential problems related to chronic kidney disease. This is necessary in order to achieve a change in behaviour and develop an appropriate existential strategy based on the idea of "dialysis as a function of life and not of a life as a function of dialysis".

Finally, in this framework the multidisciplinary team (nephrologist, psychiatrists, psychologists, nurses) is essential to plan and implement specific clinical psychological interventions and psychotherapeutic interventions to improve adherence to treatment and a mind-body integration, that is the totality of physical functioning [27,28]. The results of our study confirmed the data on the presence of psychological distress in people with chronic kidney disease on dialysis and the need for psychological support to help the patient gain a better awareness of their disease and learn new coping strategies [3,8,22,28].

The implication for future research could be the use of the CBA-H on the haemodialysis treatment. This psychological questionnaire has been used in the literature for various chronic diseases and allows us to have a greater predictive vision of the adherence of the patient to the dialysis treatment. The evaluation of the Type A personality and Vital Exhaustion syndrome are important information on mental condition that the patient is experiencing in order to support a targeted treatment. The emotional variables studied, as well as quality of life, if carefully considered and managed in the therapeutic relationship, can improve patient adherence to treatment, essential to the patient's psychological and emotional well-being.

Deepening the emotional states of subjects who experience dialysis can thus be a first important step in planning psychoeducational interventions of self-awareness and self-efficacy [29,30].

However, the small number of participants, the cross-sectional nature of the analysis, the use of self-report measures and the lack of adjustment for potential confounding variables limit the present study. Due to these limitations, this article could not provide definitive conclusions on variables that can improve lifestyle and the adherence to haemodialysis treatment. Nevertheless, it provides information and some significant evidence to support the hypothesis that the psychological well-being of dialyzed patients could be due to the combination of several factors, including life parameters, the positive perception of the psychosocial outcomes, and the perceived quality of life.

The results and limitations of the present study will be used in future work to design a new trial with a larger sample to provide further evidence the connection between psychological wellbeing and quality of life.
